# The efficacy of acupuncture for diabetic peripheral neuropathy: a systematic review and meta-analysis of randomized controlled trails

**DOI:** 10.3389/fneur.2024.1500709

**Published:** 2024-12-20

**Authors:** Ruisi Ge, Rihe Liu, Mengru He, Jiawei Wu, Feng Zhang, Chang Huang

**Affiliations:** School of Acupuncture-Moxibustion and Tuina, Beijing University of Chinese Medicine, Beijing, China

**Keywords:** acupuncture, diabetic peripheral neuropathy, nerve conduction velocity, meta-analysis, randomized controlled trails

## Abstract

**Objective:**

To systematically evaluate the clinical efficacy of acupuncture in the treatment of diabetic peripheral neuropathy (DPN).

**Methods:**

Randomized controlled trial (RCT) of acupuncture for diabetic peripheral neuropathy in Chinese Knowledge Network (CNKI), Wanfang database, VIP database (VIP), PubMed, web of science, cochrane library, AMED and CINAHL were searched by computer since the establishment of the database. All publications in English and Chinese as of 30 December 2023 will be searched, without country or article type restrictions. Study selection, data extraction and evaluation were performed independently by two researchers. Risk of bias was assessed using the Cochrane risk assessment tool, and Meta-analysis was performed using RevMan5.3 software.

**Results:**

DPN has good effective rate in acupuncture than conventional western medicine alone. However, the above conclusions need to be verified by larger samples and higher quality randomized controlled trials. ① Acupuncture treated DPN more effective than drug (RR = 1.38, 95%CI = 1.26 ~ 1.51, Z = 6.93, *p* < 0.00001), DPN of patients with acupuncture plus drug (RR = 1.38, 95%CI = 1.05 ~ 1.82, Z = 2.28, *p* = 0.02), There was no significant difference between acupuncture and usual care (RR = 2.41, 95%CI = 0.70 ~ 8.29, Z = 1.39, *p* = 0.16); ② Acupuncture treatment is superior to drug group in improving the SNCV of the median nerve (MD = 1.65, 95%CI = 0.74 ~ 2.57,Z = 3.55, *p* = 0.0004), sham needle treatment (MD = 0.50, 95%CI = 0.17 ~ 0.83, Z = 2.95, *p* = 0.003), Acupuncture plus drug was superior to drug in improving the SNCV of the median nerve (MD = 3.29, 95%CI = 2.55 ~ 4.03, Z = 8.70, *p* < 0.00001); ③ Acupuncture treatment is superior to drug group in improving the MNCV of the median nerve (MD = 2.24, 95%CI = 0.50 ~ 3.98, Z = 2.52, *p* = 0.01), and sham needle treatment (MD = 0.20, 95%CI = −0.03 ~ 0.43, Z = 1.69, *p* = 0.09), Acupuncture plus drug was superior to drug group in improving the MNCV of the median nerve (MD = 2.63, 95%CI = 1.83 ~ 3.42, Z = 6.46, *p* < 0.00001); ④ Acupuncture is better to drug group in improving SNCV of common peroneal nerve (MD = 1.67, 95%CI = 0.21 ~ 3.13, Z = 2.24, *p* = 0.02); ⑤ Acupuncture treatment is superior to drug group in improving the MNCV of the common peroneal nerve (MD = 2.03, 95%CI = 1.37 ~ 0.69, Z = 6.04, *p* < 0.00001), Acupuncture plus drug outperformed MNCV in improving the common peroneal nerve (MD = 4.23, 95%CI = −0.16 ~ 8.62, Z = 1, 89, *p* = 0.06); ⑥ Acupuncture treatment is superior to drug group in improving the SNCV of the tibial nerve (MD = 1.58, 95%CI = 0.85 ~ 2.30, Z = 4.26, *p* < 0.0001); ⑦ There was no significant difference between acupuncture treatment and drug group in improving the MNCV of the tibial nerve (MD =1.55, 95%CI = −0.59 ~ 3.68, Z = 1.42, *p* = 0.16); ⑧ Acupuncture plus drug is better than medication in reducing VAS (MD = −2.35, 95%CI = -3.78 ~ −0.93, Z = 3.23, *p* = 0.001), Acupuncture plus usual care is superior to usual caret (MD = −28.70, 95%CI = -39.50 ~ 17.90, Z = 5.21, *p* < 0.00001), There was no significant difference between acupuncture and sham needle treatment (MD = −4.00, 95%CI = -18.32 ~ 10.32, Z = 0.55, *p* = 0.58).

**Conclusion:**

Compared with drug, usual care, and sham AT, AT has a better response rate and more favorable effect in improving nerve conduction velocity. The combination of AT and drug demonstrates a more significant improvement compared to drug alone. The combination of AT and usual care improves DPN symptoms more effectively than usual care. However, the above conclusions need to be verified by larger samples and higher quality randomized controlled trials.

**Systematic review registration:**

[https://www.crd.york.ac.uk/], identifier [CRD42023451575].

## Graphical abstract

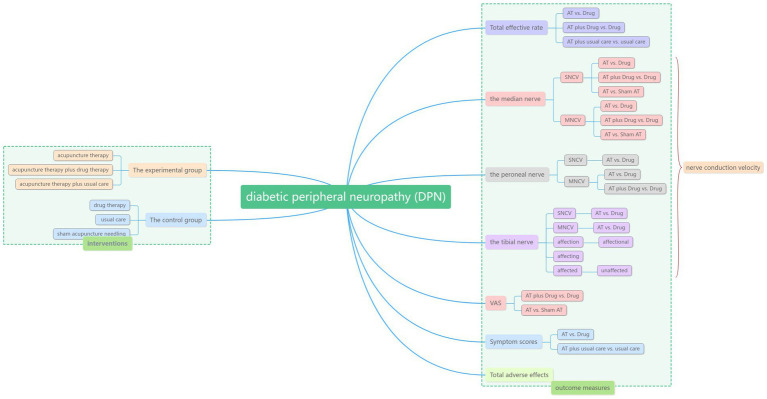

## 1 Introduction

Diabetes mellitus is a metabolic disease characterized by hyperglycaemia that affects a large population, is highly dangerous and is difficult to treat (1). Peripheral neuropathy (DPN) is one of the most common complications in people with diabetes. It is characterized by numbness, pain, burning or other abnormal sensations in the limbs. The WHO predicts that by 2030, there will be approximately 360 million diabetic patients worldwide, and more than 50% of them may have DPN symptoms, and most diabetic amputations or disabilities are caused by DPN (2, 3), and the quality of life of diabetic patients with DPN will be seriously reduced once it occurs, and even lead to the death of the patients. Studies have shown that the relative likelihood of death within 5 years of lower limb amputation due to diabetic foot ulcers is greater than for diseases such as prostate and breast cancer (4). In addition, amputation imposes a significant financial burden on both the healthcare system and society. In the United States, the total annual cost of care for symptomatic DPN (pain) and its complications (foot ulcers and lower limb amputations) is estimated to be between 460 million and 1,370 million US dollars. As much as 27% of the direct medical costs associated with diabetes are attributed to DPN (5).

Diabetic peripheral neuropathy is usually irreversible. The medical community does not have a consistent and effective treatment plan to manage the disease. Treatment options at this stage are generally used to prevent disease transmission and complications. Most treatment options tend to use symptomatic treatment such as nerve nutrition and improvement of neural microcirculation (6). Commonly used drugs include alpha-lipoic acid, which is an antioxidant stress, neurotrophins such as vitamin B1, B12, gangliosides, nerve growth factor, etc., and drugs to improve neural microcirculation include prostaglandin E1, scopolamine and hexacosanolone coccolithophore, etc. (7). Drugs are mainly used to relieve neuropathic pain and sensory abnormalities, but they cannot solve the problem of decreased nerve function. There is still a lack of specific therapeutic measures, and there are no effective treatments and medications, and most of the medications have certain side effects, and patients must be able to tolerate the side effects of drug therapy (8, 9). In addition, there are individual differences between patients, and western medical treatment has more adverse effects and poor long-term results (10).

Acupuncture is a characteristic external treatment method of traditional Chinese medicine, which mainly plays a therapeutic role through various physical stimulation effects on acupoints. Nowadays, this therapy has been widely used in the treatment of diabetic complications, including diabetic foot, diabetic bladder, diabetic peripheral neuropathy and so on. From the clinical study report, acupuncture can significantly reduce the symptoms of numbness, pain and superficial sensory impairment of the extremities in patients with DPN, with certain efficacy and fewer side effects (11). In addition, acupuncture has the advantages of multi-targeting and bidirectional regulation of the mode of action (12). It is currently believed that the pathological mechanism of DPN is closely related to inflammation, oxidative stress, endoplasmic reticulum stress, microvascular lesions, neurotrophic disorders and immune dysfunction (13), and its pathological changes are peripheral nerve demyelination or axonal degeneration (14), or both. Acupuncture has the ability to modulate inflammatory reaction, oxidative stress, ER stress, increase peripheral nerve blood flow, ameliorate microangiopathy, increase neurotrophic factor content, ameliorate peripheral nerve electrophysiological function, promote axonal and myelin repair, and so on. The key to the therapeutic effects of acupuncture in DPN may be found in the above mechanisms (15).

A recent systematic review of acupuncture for the treatment of diabetic peripheral neuropathy concluded that acupuncture can effectively improve the neurological and clinical symptoms of diabetic peripheral neuropathy, but further work is needed to develop a uniform standard for the treatment of diabetic peripheral neuropathy with acupuncture (16). Although we also use acupuncture for the treatment of diabetic peripheral neuropathy in the clinic, there is a lack of systematic data studies on the efficacy of acupuncture for the treatment of diabetic peripheral neuropathy. Therefore, a meta-analysis was performed in this paper to summarize the randomized controlled trials of acupuncture for diabetic peripheral neuropathy published by previous investigators.

## 2 Methods

### 2.1 Literature search strategy

The search will include major Chinese and English databases such as PubMed, Web of Science, Cochrane Library, AMED, CINAHL, China Knowledge Network (CNKI), Wanfang Database and Wipro Database (VIP), supplemented by references to included trials, clinical trial or research registry platforms, expert consultation and gray literature. All publications in Chinese and English from the time of the library’s inception to 30 December 2023 will be searched, regardless of country or article type. The key search terms were composed of the following group terms: “acupuncture,” “acupuncture,” “electropuncture,” “fire needle,” “plum blossom needle,” “acupoint,” “auricular acupuncture” and “peripheral nervous system diseases,” “diabetic peripheral neuropathy,” “DPN” and “randomized controlled trial,” “RCT,” “random,” “blind,” “control.”

### 2.2 Inclusion criteria

The studies which were included must meet the following eligibility criteria.

#### 2.2.1 Type of trials

English and Chinese RCTs, excluding trials, case studies, case series, qualitative studies and uncontrolled studies. Trials that did not report detailed outcomes were also excluded, and there were no restrictions on time to publication or geographical location of the study.

#### 2.2.2 Type of participants

Patients meeting the diagnostic criteria for DPN or using self-developed Chinese or Western diagnostic criteria. There were no restrictions on baseline information such as sex, age, race and region of patients, but they must be comparable.

#### 2.2.3 Type of interventions

The experimental group was acupuncture therapy, acupuncture therapy plus drug therapy, or acupuncture therapy plus usual care.

The control group was drug therapy (Details of the intervention have been descripted in Table 1), sham acupuncture needling, or usual care (Maintain blood sugar within the normal range of fasting, without using any other therapeutic approaches).

**Table 1:** Baseline characteristics of included studies.

	Number	Study author and year	Grouping method	Sample size (Male/Female)		Mean age or age range, years		Intervention measure		Treatment periods	Outcome measures	Authors conclusion	Adverse effects
				Intervention group	Comparison group	Intervention group	Comparison group	Intervention group	Comparison group				
AT VS drug	1	Zhou 2018	Random number table method	30(16/14)	30(15/15)	54.32 ± 8.23	56.75 ± 7.46	(A) AT (without retention, once per 2 days or once per 3 times weekly for 30 days, *n* = 30)	(B) Drug therapy (Mecobalamin, oral, 0.5 mg, 3 times daily for 30 days, *n* = 30)	30 days	①(1/2/3/4)②④	“Acupuncture therapy can not only alleviate ……, but also effectively improve ……, and other advantages.”	Not given
	2	Jiang 2005	Randomized	30(17/13)	30(21/9)	61.6 ± 12.08	60.8 ± 11.47	(A) AT (15 ~ 30 min, once daily for 21 days, *n* = 30)	(C) Drug therapy (VitB1, VitB12, injection, once daily for 21 days)	21 days	①(2/4)②	“Acupuncture therapy can improve……, …… can be restored.”	Not given
	3	Sun 2008	Randomized	26(15/11)	26(17/9)	61.00 ± 9.27	60.70 ± 6.56	(A) AT(20 min, six times weekly for 28 days, *n* = 26)	(B) Drug therapy (Mecobalamin, injection, 500 μg, once daily for 28 days, *n* = 26)	4 weeks	①(4/5)②	“Acupuncture has good clinical efficacy and can improve……”	Not given
	4	Fei 2011	Random number table method	30(17/13)	30(19/11)	54 ± 1	55 ± 1	(A) AT (30 min, once daily for 30 days, *n* = 30)	(B) Drug therapy (Mecobalamin, oral, 500 μg,3 times daily for 30 days, *n* = 30)	30 days	①(5/6)②	“Acupuncture can effectively …… improve ……”	Not given
	5	Dong 2003	Randomized	67	31	59.2 ± 6.72		(A) AT(30 min, once per 2 days for 2 months, *n* = 67)	(B) Drug therapy(VitB1, injection, 100 mg, once per 2 days for 2 months, VitB12, injecteion, 500 μg, once per 2 days for 2 months, *n* = 31)	2 months	① (3)②④⑤	“Acupuncture can improve ……, and the effect is better than ……”	Mild dizziness during acupuncture treatment (1)
	6	Lu 2016	Random number table method	31(20/11)	29(17/12)	66 ± 7	64 ± 7	(A) AT(30 min, once daily for 30 days, *n* = 31)	(B) Drug therapy (Thioctic acid injection,0.6 g, Alprostadil,10 mg, injection, once daily for 30 days, *n* = 29)	30 days	①(1/2/3/4)②④	“Acupuncture treatment has a better effect ……, restoring ……,alleviating ……”	Small hematomas (not given)
	7	Song 2005	Random number table method	22(9/13)	20(7/13)	58.92 ± 5.24	58.92 ± 5.24	(A) AT(20 ~ 30 min, once daily for a month, *n* = 22)	(B) Drug therapy (VitB1,100 mg, VitB12, 500 μg, injection, three times weekly for a month; VitB1, 20 mg, VitB6, 20 mg, oral, three times daily for a month, *n* = 20)	1 month	②	“Acupuncture can improve……”	Not given
	8	Zhao 2007	Randomized	30(16/14)	30(16/14)	62.30 ± 7.33	62.17 ± 7.93	(A) AT(30 min, once per 2 days for 2 months, *n* = 30)	(B) Drug therapy (Mecobalamin, 500 μg, oral, three times daily for 2 months)	2 months	①(5/6)②	“Acupuncture can improve……, is better than……, can improve clinical efficacy.”	Not given
	9	He 2005	Randomized	42(20/22)	36(16/20)	55.3 ± 2.6	53.9 ± 1.9	(A) AT (30 min, once daily for 30 days, *n* = 42)	(B) Drug therapy (Mecobalamin, oral, 500 μg, three times daily for 30 days; VitB1, injection,100 mg, VitB12, injection, 0.5 mg, once daily for 30 days, *n* = 36)	30 days	①(1/4/6)②	“Acupuncture can also improve……, increase……, and alleviate ……”	Not given
	10	Li 2000	Randomized	48	36	63.03 ± 9.84		(A) AT (30 min,3 times weekly for 2 months, *n* = 48)	(B) Drug therapy (Mecobalamin, oral, 500 μg, 3 times daily for 2 months, *n* = 36)	2 months	②④	“Acupuncture can relieve ……,improve ……, and is an effective method …….”	Not given
	11	Li^a 2021	Random number table method	Acupuncture group:31(18/13)	Drug group:31(17/14)	Acupuncture group:59.0 ± 8.0	Drug group:57.0 ± 8.0	(A) AT(30 min, once daily for 18 days, *n* = 31)	(B) Drug therapy (lipoic acid injection, injection,0.6 g, once daily for 18 days, mecobalamin, injection, 0.5 mg, once daily for 18 days /mecobalamin, oral, 0.5 mg, 3 times for 18 days)	18 days	①(1/2/3/4)②	“Acupuncture ……increasing the nerve conduction, promoting …….”	Not given
AT +drug VS drug		Li^b 2021	Random number table method	Drug and acupuncture group:31(18/13)	drug group:31(17/14)	Drug and acupuncture group:56.0 ± 9.0	Drug group:57.0 ± 8.0	(A) AT(30 min, once daily for 18 days, n = 31), plus B	(B) Drug therapy (lipoic acid injection, injection,0.6 g, once daily for 18 days, mecobalamin, injection,0.5 mg, once daily for 18 days/mecobalamin, oral, 0.5 mg, 3 times for 18 days)	18 days	①(1/2/3/4)②	“The effect of acupuncture combined drug was better than ……”	Not given
	12	Liu 2011	Random number table method	32(14/18)	32(17/15)	54.32 ± 9.72	63.78 ± 10.13	(A) AT (30 min, once daily for 12 weeks, n = 32). Moxibustion (15 min, once daily for 12 weeks, *n* = 32), plus B	(B) Drug therapy (0.9%NaCl,100 mL and Shen fu injection 50 mL, injection, once daily for 12 weeks; mecobalamin, oral,0.5 mg,3 times daily for 12 weeks, *n* = 32)	12 weeks	①(2/4)②	“Acupuncture treatment are significantly improved ……,superior to drug therapy alone.”	No obvious adverse reactions occurred
	13	Deng 2021	Random number table method	30(18/12)	30(15/15)	59 ± 9	57 ± 9	(A) AT (30 min, once daily for 4 weeks, *n* = 30)	(B) Drug therapy (Pregabalin Capsules, oral, 75 mg, twice daily for 4 weeks, *n* = 30)	4 weeks	①(1/2/5/6)②③	“Dragon-tiger acupuncture can effectively improve ……, enhance……”	Not given
	14	Shu 2021	Random number table method	30(15/15)	30(19/11)	64 ± 8	65 ± 7	(A) AT(20 min,3 times weekly for 4 weeks, *n* = 30)	(B) Drug therapy (mecobalamin, oral, 0.5 mg, three times daily for 4 weeks. n = 30)	4 weeks	②③	“Acupuncture ……, can improve ……, safety ……”	Tiredness (1)
AT VS sham AT	15	Tong 2010	Random number table method	42(9/33)	21(5/16)	43.5 ± 5.0	45.8 ± 6.2	(A) AT(30 min, once daily for 15 days, n = 42)	(B) Sham AT(30 min, once daily for 15 days, n = 21)	15 days	①(1/2/6)	“Acupuncture …… improved nerve conduction velocity …… subjective symptoms ……”	Not given
	17	Wang 2020	Random number table method	105	105	Not given	Not given	(A) AT(30 min, 3 times weekly for 6 weeks, *n* = 105)	(B) Sham AT(30 min, 3 weekly for 6 weeks, *n* = 105)	6 weeks	① (6)④	“Acupuncture is effective……”	Not given
	18	Garrow 2014	Computer-generated randomization list method	24(16/8)	21(15/6)	68 ± 11.1	63 ± 10.8	(A) AT(30 min, once weekly for 10 weeks, n = 24)	(B) Sham AT(30 min, once weekly for 10 weeks, n = 21)	10 weeks	③⑤	“Acupuncture …… successfully incorporated ……”	Chest pains (1) pain exacerbated (1) localized swelling (1)
AT + usual care VS usual care	19	Dietzel 2023	Computer-generated randomization list method	31(25/6)	31(24/7)	66.7 ± 7.6	69.5 ± 7.2	(A) AT(25 min, 12 times for 8 weeks, n = 31), plus B	(B) Usual care (8 weeks, n = 31)	8 weeks	③⑤	“Acupuncture…… significant and lasting reductions ……”	Small hematomas (18) transient paraesthesia (7) transient pain (5) tiredness (5) transitory intensifying of DPN- related symptoms (4) cramps (1) light- headed (1) itching (1)
	20	Xu 2003	Computer-generated randomization list method	34(15/19)	28(11/17)	53.62 ± 11.17	54.24 ± 13.54	(A) AT (30 min, once per 2 days for 20 days, n = 34), plus B	(B) Usual care (20 days, *n* = 28)	20 days	②	“Acupuncture …… has obtained obvious curative effect”	Not given
	21	Wang 2013	Random number table method	41(21/20)	41(17/24)	77.5 ± 4.3	81.2 ± 2.1	(A) AT (30 min, 6 times weekly for 3 weeks, *n* = 41), plus B	(B) Usual care (3 weeks, *n* = 41)	3 weeks	②④	“Acupuncture ……achieve good therapeutic effect.”	Not given

①, Nerve conduction velocity (1 SNCV in median nerve 2 MNCV in median nerve, 3 SNCV in common peroneal nerve, 4 MNCV in common peroneal nerve, 5 SNCV in tibial nerve, 6 MNCV in tibial nerve); ②, effective rate; ③, VAS; ④, symptom score; ⑤, adverse effects.

#### 2.2.4 Type of outcome measures

Treatment efficacy; sensory nerve conduction velocity (SNCV) of the median, common peroneal and tibial nerves; motor nerve conduction velocity (MNCV) of the median, common peroneal and tibial nerves; and visual pain scale (VAS); Symptom scores.

### 2.3 Exclusion criteria

The following were excluded: duplicate publications; unavailability of original literature; literature with incomplete or questionable data; non-RCT literature such as case studies, case series, qualitative studies and uncontrolled trials; comorbidities with other causes of peripheral neuropathy; and patients in the study group who had received other TCM drugs or therapies during the course of their disease.

### 2.4 Studies selection and data extraction

The literature was screened independently by two researchers in accordance with the inclusion and exclusion criteria and the literature search strategy, and the retrieved literature was imported into Endnote20 software for duplicate checking and removal. The title and abstract were read first to exclude literature that clearly did not meet the inclusion criteria. The remaining literature was read in full and screened again to identify literature that met the inclusion criteria. In the case of conflicting opinions, the third researcher was asked to participate in the discussion for assessment.

The extraction of data was conducted independently by two investigators, encompassing fundamental details pertaining to the study, such as title, author, publication date, and journal, alongside essential study characteristics, including mean age, gender, sample size, subgroups, measures, treatment duration, follow-up duration, and outcome measures.

The quality of the included trials was assessed by the researchers using the risk of bias assessment tool recommended in the Cochrane Handbook for Systematic Evaluators, and the results of the assessment included the following six items: whether the random allocation method was appropriate, whether allocation concealment was correctly applied, whether blinding was correctly applied, whether there was no selective reporting of results, whether outcome data were complete, whether there was any other risk of bias.

### 2.5 Statistical analysis

Meta-analysis was performed with the use of RevMan 5.3 software. For dichotomous variables (e.g., clinical effectiveness), the relative risk (RR) was used as the effect size, and for continuous variables (e.g., sensory nerve conduction velocity SNCV), the mean difference (MD) was used as the effect size, and the 2 effect sizes were expressed by 95% CI. The degree of heterogeneity was determined by the I2 and *p* values; when the heterogeneity between studies was small (I2 ≤ 50%, *p* > 0.05), a fixed-effects model was chosen; when heterogeneity between studies was present (I2 ≥ 50%, *p* < 0.05), a random-effects model was chosen. A p value less than 0.05 signifies a statistically significant difference.

### 2.6 Sensitivity analysis

Sensitivity analysis was performed to verify the robustness of the results of the heterogeneity tests by excluding studies case-by-case. In addition, the Baujat plot was used to further characterize the contribution of each study to overall heterogeneity and identify high heterogeneity studies.

### 2.7 Publication bias

If more than 10 studies were available, publication bias was assessed using funnel plots. In addition, Egger test or Peters test was used to further formally test for potential publication bias.

## 3 Results

### 3.1 Search results

**Figure 1:**
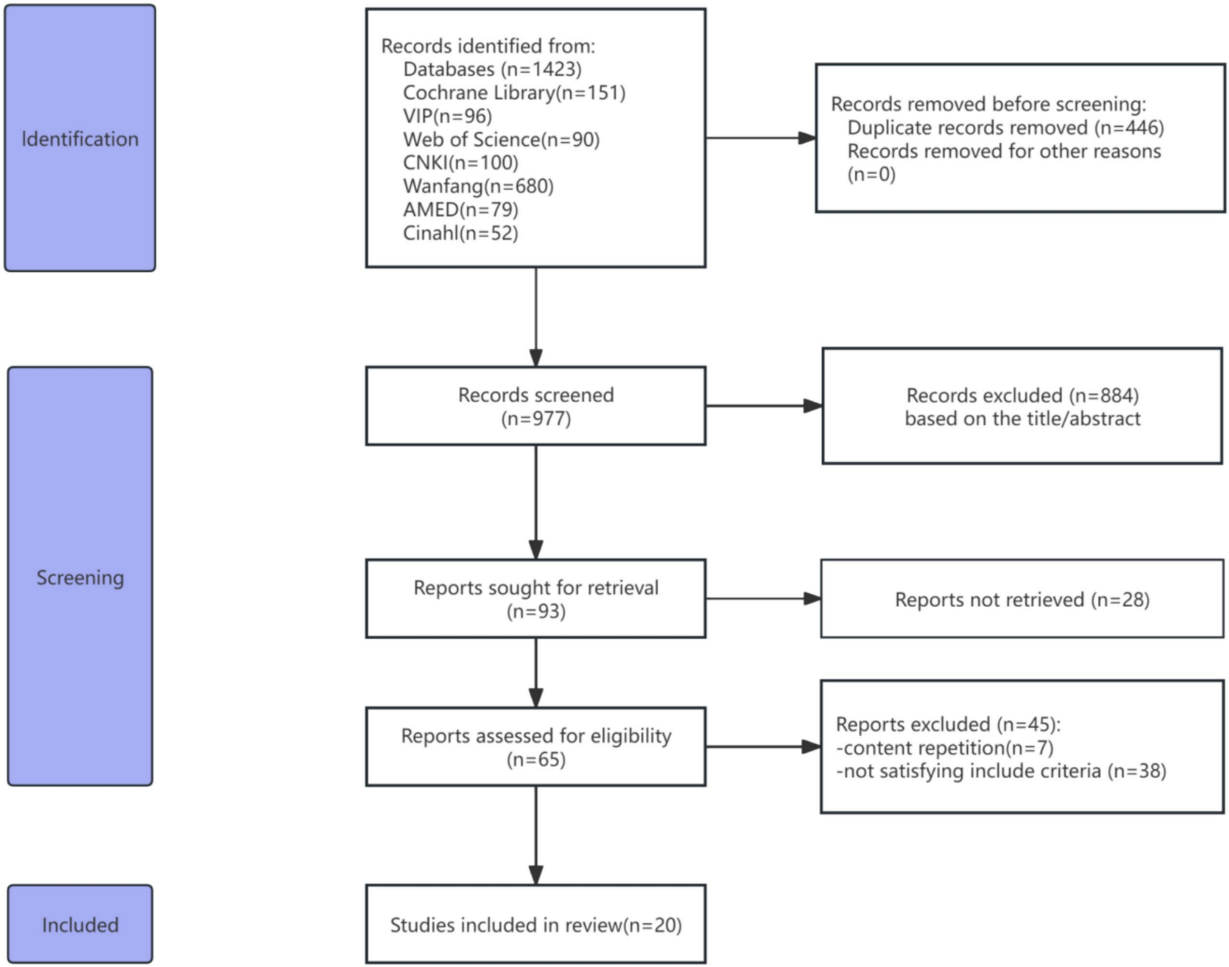
PRISMA flowchart of the included studies.

A total of 1,423 studies were identified in eight databases. A total of 446 articles were removed due to duplication. A total of 884 studies were screened by reading titles and abstracts, leaving 93 articles. After reading the full text of 93 articles, 73 articles were excluded for the reasons described in Figure 1. Finally, 20 studies (17–36) met the inclusion criteria and were meta-analyzed (Figure 1).

### 3.2 Study characteristics

A total of 20 papers were included in this review, all of which were published, including 6 in English (17–20, 26, 36) and 14 in Chinese (21–25, 27–35), involving a total of 1,455 patients. All studies reported comparable baseline data between groups. All trials included adults, and the mean age of participants ranged from 45 to 81 years, with all being middle-aged to older adults. Eleven trials compared AT with medication (19, 21, 23–27, 31–34), and four trials used AT + medication as an intervention and medication alone as a control (22, 26, 30, 35), with one trial having two intervention groups: AT and AT plus medication (26). Three trials compared AT with sham AT (18, 20, 36). Three trials compared AT + conventional treatment with conventional treatment (17, 28, 29).

### 3.3 Risk of bias assessment

**Figure 2:**
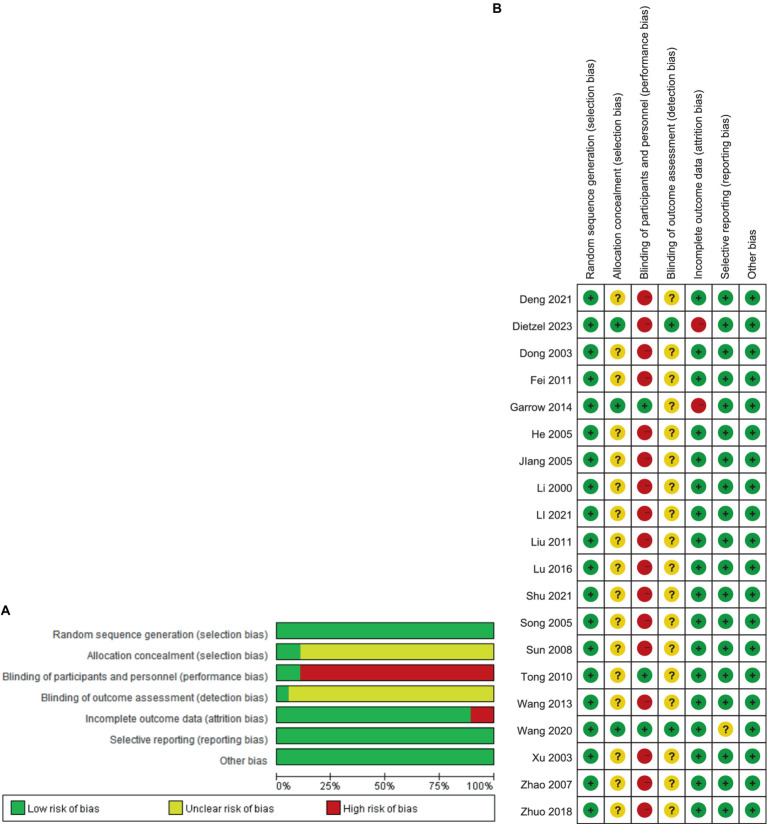
Risk of bias from included studies. **(A)** Risk of bias graph. **(B)** Risk of bias summary.

The results of the risk of bias assessment are displayed in Figure 2. According to the Cochrane Risk of Bias Assessment Tool, 20 studies mentioned randomization and all of the literature used the random grouping method, of which 11 articles used the random number table method (20, 22, 23, 25, 26, 28, 30, 32, 34–36), three used the method of using computer random grouping (17, 18, 29), and the rest of the articles did not mention the specific random grouping method; one study (18) mentioned allocation concealment by using sealed opaque envelopes, one study (17) had randomization performed by a single research nurse and notified the study doctors and patients of the allocation results by telephone, and the rest did not describe the method of allocation concealment; 2 studies (18, 20) blinded patients using a sham-needle technique, the remaining studies referred to in-process blinding during the trial, and blinding of patients or clinicians was considered to be at high risk due to the variable differences between the intervention and control groups; 1 study (17) referred to blinding of data analysts, the remaining studies did not; 2 studies (17, 18) reported off-case results, the remaining studies did not report off-case results, with good data completeness; no selectivity was reported in all studies, with a low risk of other biases.

### 3.4 Effects of interventions

#### 3.4.1 Total effective rate

**Figure 3:**
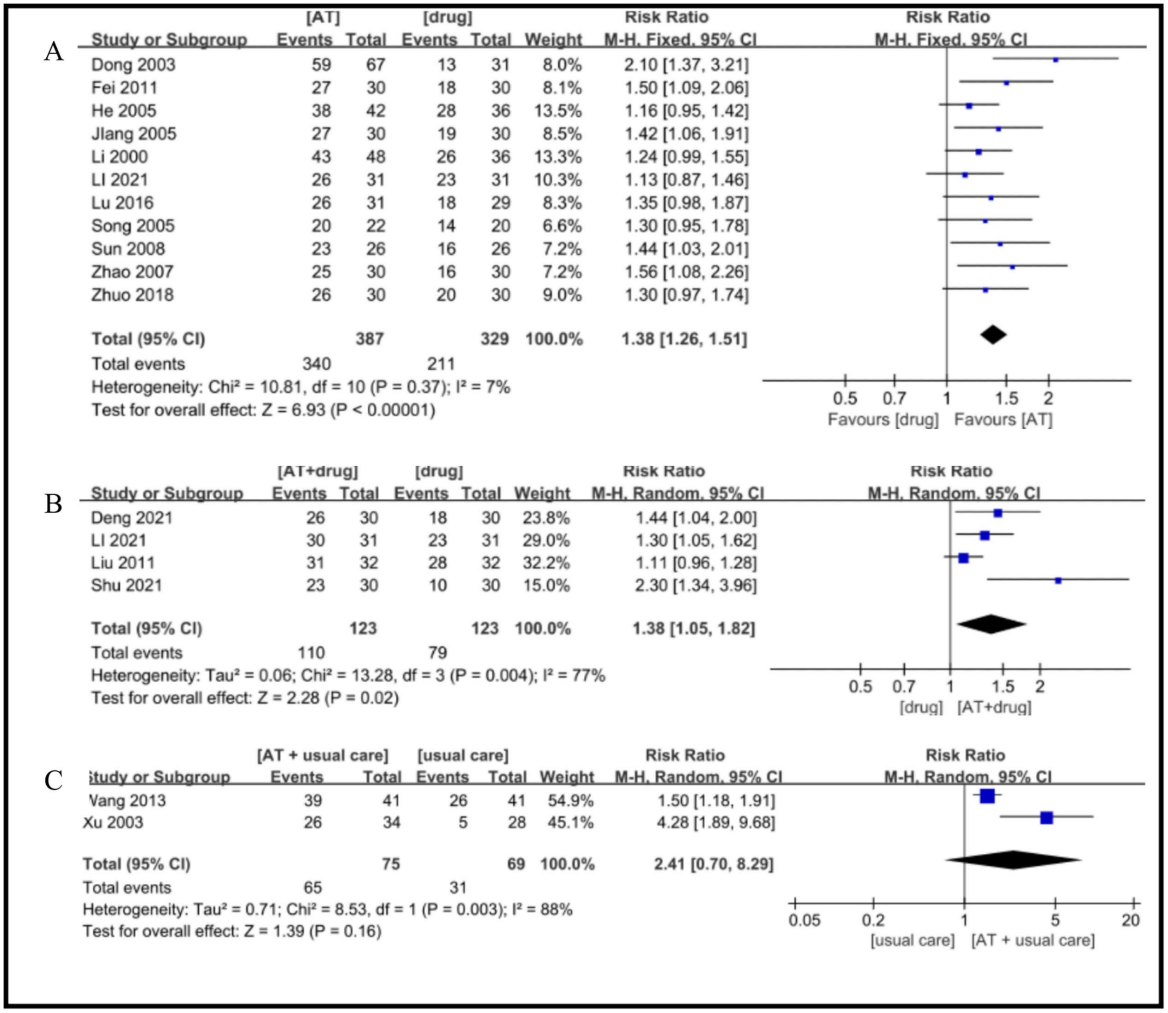
A Forest plot for total effective rate according to the comparison of **(A)** AT vs. Drug, **(B)** AT plus Drug vs. Drug, **(C)** AT plus usual care vs. usual care.

Eleven RCTs were included (19, 21, 23–27, 31–34), and the heterogeneity test showed that the heterogeneity between studies was small (I^2^ = 7%, *p* = 0.37), and a fixed-effects model was adopted. The results of the meta-analysis showed that the overall efficacy rate of patients treated with acupuncture was significantly better than that of patients treated with drugs, and the difference was statistically significant (RR = 1.38, 95%CI = 1.26 ~ 1.51, Z = 6.93, *p* < 0.00001, Figure 3A).

Four RCTs were included (22, 26, 30, 35). Heterogeneity analysis revealed substantial heterogeneity among the studies (I^2^ = 77%, *p* = 0.004), necessitating the application of a random-effects model. The meta-analysis demonstrated that the combined use of acupuncture and drug therapy yielded a higher overall efficacy rate compared to drug therapy alone, with a statistically significant difference (RR = 1.38, 95% CI = 1.05–1.82, Z = 2.28, *p* = 0.02, Figure 3B).

Two RCTs were included (28, 29), with a high level of heterogeneity observed (I^2^ = 88%, *p* = 0.003), prompting the adoption of a random-effects model. The meta-analysis revealed no significant difference in the overall efficacy rate between patients treated with AT plus usual care and those receiving usual care alone (RR = 2.41, 95%CI = 0.70 ~ 8.29, Z = 1.39, *p* = 0.16, Figure 3C).

#### 3.4.2 SNCV of the median nerve

**Figure 4:**
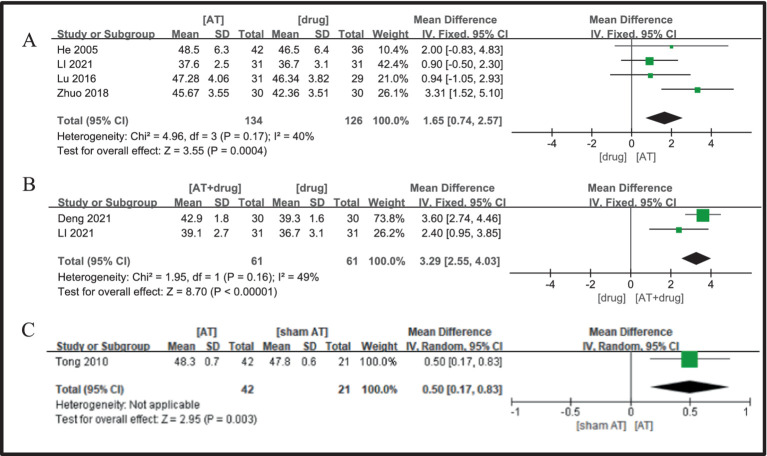
A Forest plot for SNCV of the median nerve according to the comparison of **(A)** AT vs. Drug, **(B)** AT plus Drug vs. Drug, **(C)** AT vs.sham AT.

Four studies (21, 23, 26, 34) were analyzed, demonstrating low heterogeneity (I^2^ = 40%, *p* = 0.17), thus supporting the use of a fixed-effects model. The meta-analysis indicated that patients in the acupuncture group experienced a statistically significant enhancement in median sensory nerve conduction velocity, compared to the drug group, with a mean difference of 2.61 (MD = 1.65, 95%CI = 0.74 ~ 2.57, Z = 3.55, *p* = 0.0004, Figure 4A).

Two studies were included (26, 35), and the heterogeneity analysis indicated minimal inter-study variability (I^2^ = 49%, *p* = 0.16), leading to the adoption of a fixed-effects model. The analysis demonstrated that the protocol combining acupuncture and drug was superior to drug alone in enhancing median nerve sensory nerve conduction velocity in patients with diabetic peripheral neuropathy, with the difference being statistically significant (MD = 3.29, 95%CI = 2.55 ~ 4.03, Z = 8.70, *p* < 0.00001, Figure 4B).

In a study (20), the protocol for the acupuncture treatment group was found to outperform the sham needle group in improving sensory nerve conduction velocity in the median nerve of patients with diabetic peripheral neuropathy (DPN). This superiority was accompanied by a statistically significant increase in median nerve conduction velocity, indicating a notable difference in the treatment outcomes (MD = 0.50, 95%CI = 0.17 ~ 0.83, Z = 2.95, *p* = 0.003, Figure 4C).

#### 3.4.3 MNCV of the median nerve

**Figure 5:**
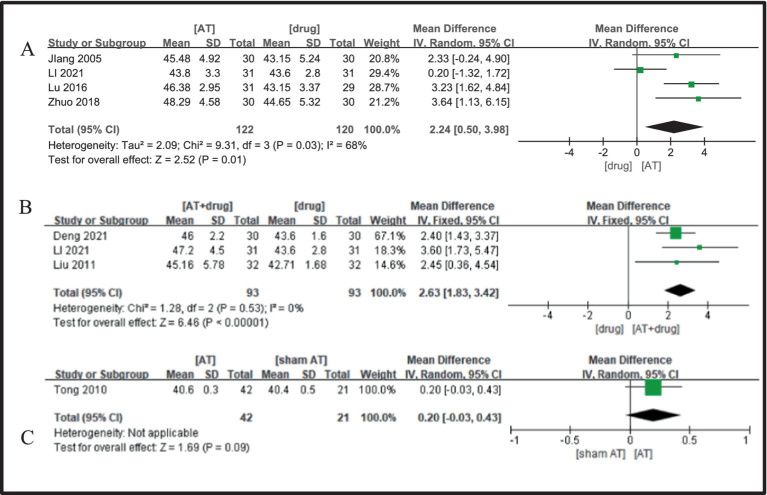
A Forest plot for MNCV of the median nerve according to the comparison of **(A)** AT vs. Drug, **(B)** AT plus Drug vs. Drug, **(C)** AT vs.sham AT.

Four studies (19, 23, 26, 34) were included, showing no significant heterogeneity (I^2^ = 68%, *p* = 0.03), thus justifying the use of a fixed-effects model. The comparison revealed that the acupuncture treatment group outperformed the drug treatment group in improving motor nerve conduction velocity of the median nerve in patients with diabetic peripheral neuropathy, with the observed difference being statistically significant (MD = 2.24, 95%CI = 0.50 ~ 3.98, Z = 2.52, *p* = 0.01, Figure 5A).

Three studies (22, 26, 35) were analyzed, revealing negligible between-study heterogeneity (I^2^ = 0%, *p* = 0.53), which supported the utilization of a fixed-effects model. The meta-analysis outcomes demonstrated that the acupuncture plus medication group exhibited superiority over the medication group in improving motor nerve conduction velocity of the median nerve in diabetic peripheral neuropathy patients, with a statistically significant discrepancy noted (MD = 2.63, 95%CI = 1.83 ~ 3.42, Z = 6.46, *p* < 0.00001, Figure 4B).

One study was included (20), and the meta-analysis outcomes indicate that the acupuncture group protocol does not exhibit a significant difference compared to the sham needle group in enhancing the motor nerve conduction velocity of the median nerve in patients suffering from DPN (MD = 0.20, 95%CI = −0.03 ~ 0.43, Z = 1.69, *p* = 0.09, Figure 5C).

#### 3.4.4 SNCV of the peroneal nerve

**Figure 6:**
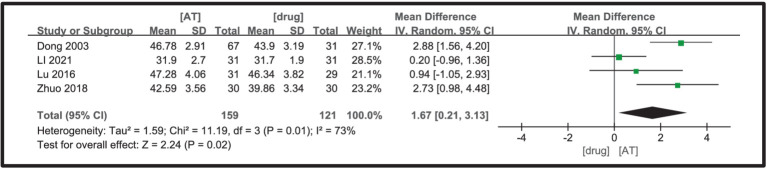
A forest plot for SNCV of the peroneal nerve.

Four studies (23, 26, 31, 34) were incorporated, and the heterogeneity analysis revealed minimal between-study variability (I^2^ = 73%, *p* = 0.01), thus justifying the use of a fixed-effects model. The meta-analysis outcomes indicated that the acupuncture treatment group outperformed the drug treatment group in enhancing the sensory nerve conduction velocity of the common peroneal nerve in diabetic peripheral neuropathy (DPN) patients, with a statistically significant difference observed (MD = 1.67, 95%CI = 0.21 ~ 3.13, Z = 2.24, *p* = 0.02, Figure 6).

#### 3.4.5 MNCV of the peroneal nerve

**Figure 7:**
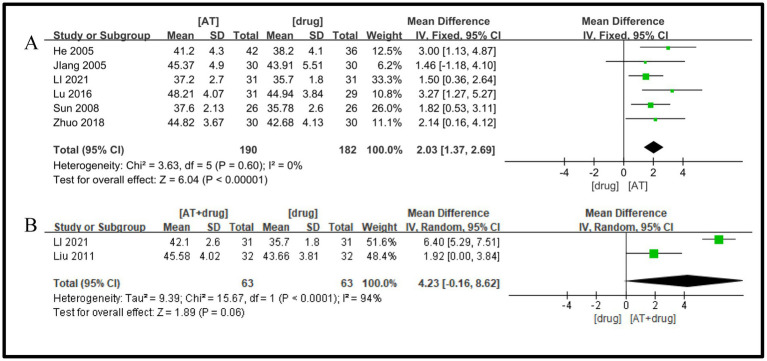
A Forest plot for MNCV of the peroneal nerve according to the comparison of **(A)** AT vs. Drug, **(B)** AT plus Drug vs. Drug.

Six studies (19, 21, 23, 24, 26, 34) were included, and a significant level of inter-study heterogeneity was detected (I^2^ = 0%, *p* = 0.60), prompting the adoption of a random-effects model. The comparison indicated that the acupuncture treatment group outperformed the drug treatment group in enhancing the motor nerve conduction velocity of the common peroneal nerve in diabetic peripheral neuropathy (DPN) patients, with a statistically significant difference (MD = 2.03, 95%CI = 1.37 ~ 2.69, Z = 6.04, *p* < 0.00001, Figure 7A).

Two studies (22, 26) were included, with a substantial heterogeneity found between them (I^2^ = 94%, *p* < 0.0001), prompting the use of a random effects model. The meta-analysis did not identify a statistically significant difference between the protocols of the acupuncture plus medication group and the medication group in terms of improvement in motor nerve conduction velocity of the common peroneal nerve in patients with diabetic peripheral neuropathy (DPN) (MD = 4.23, 95%CI = −0.16 ~ 8.62, Z = 1.89, *p* = 0.06, Figure 7B).

#### 3.4.6 SNCV of the tibial nerve

**Figure 8:**
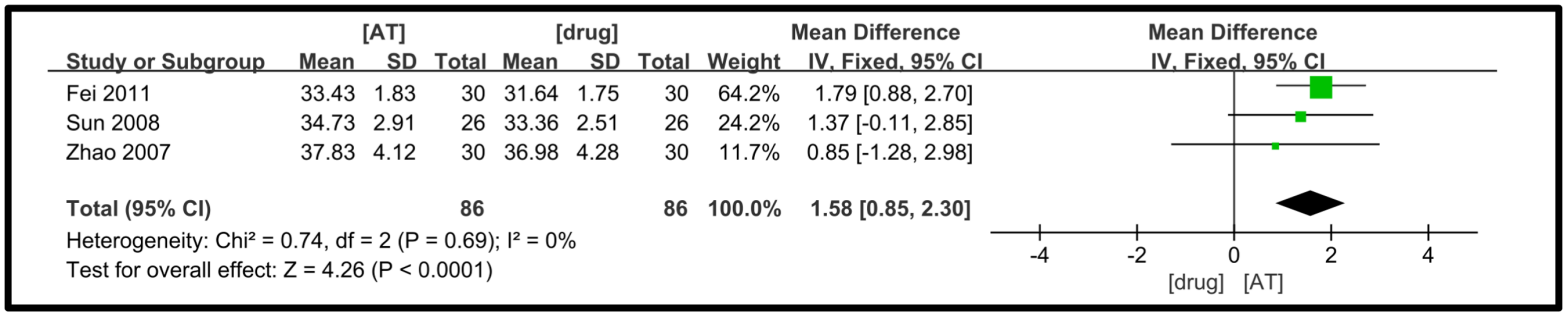
A forest plot for SNCV of the tibial nerve.

Three studies (24, 32, 33) were examined, and the heterogeneity test indicated a negligible degree of between-study heterogeneity (I^2^ = 0%, *p* = 0.69), prompting the utilization of a fixed-effects model. The meta-analysis findings revealed that the acupuncture treatment group exhibited superiority over the drug treatment group in enhancing the sensory nerve conduction velocity of the common peroneal nerve in DPN patients, with a statistically significant difference detected (MD = 1.58, 95%CI =0.85 ~ 2.30, Z = 4.26, *p* < 0.0001, Figure 8).

#### 3.4.7 MNCV of the tibial nerve

**Figure 9:**
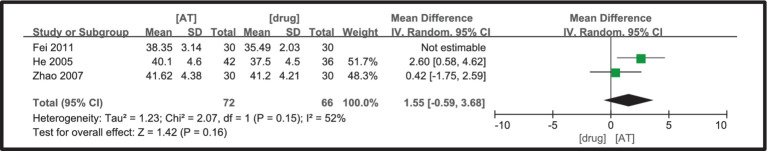
A forest plot for MNCV of the tibial nerve.

Three studies were included (21, 32, 33), and the heterogeneity test showed that there was a large heterogeneity between studies (I^2^ = 52%, *p* = 0.15), and a random-effects model was adopted. All three studies showed that, compared with medication, the acupuncture group had a statistically significant effect on the improvement of motor nerve conduction velocity of the tibial nerve in DPN patients (MD = 1.55, 95% CI = −0.59 ~ 3.68, Z = 1.42, *p* = 0.16, Figure 9).

#### 3.4.8 Vas

**Figure 10:**
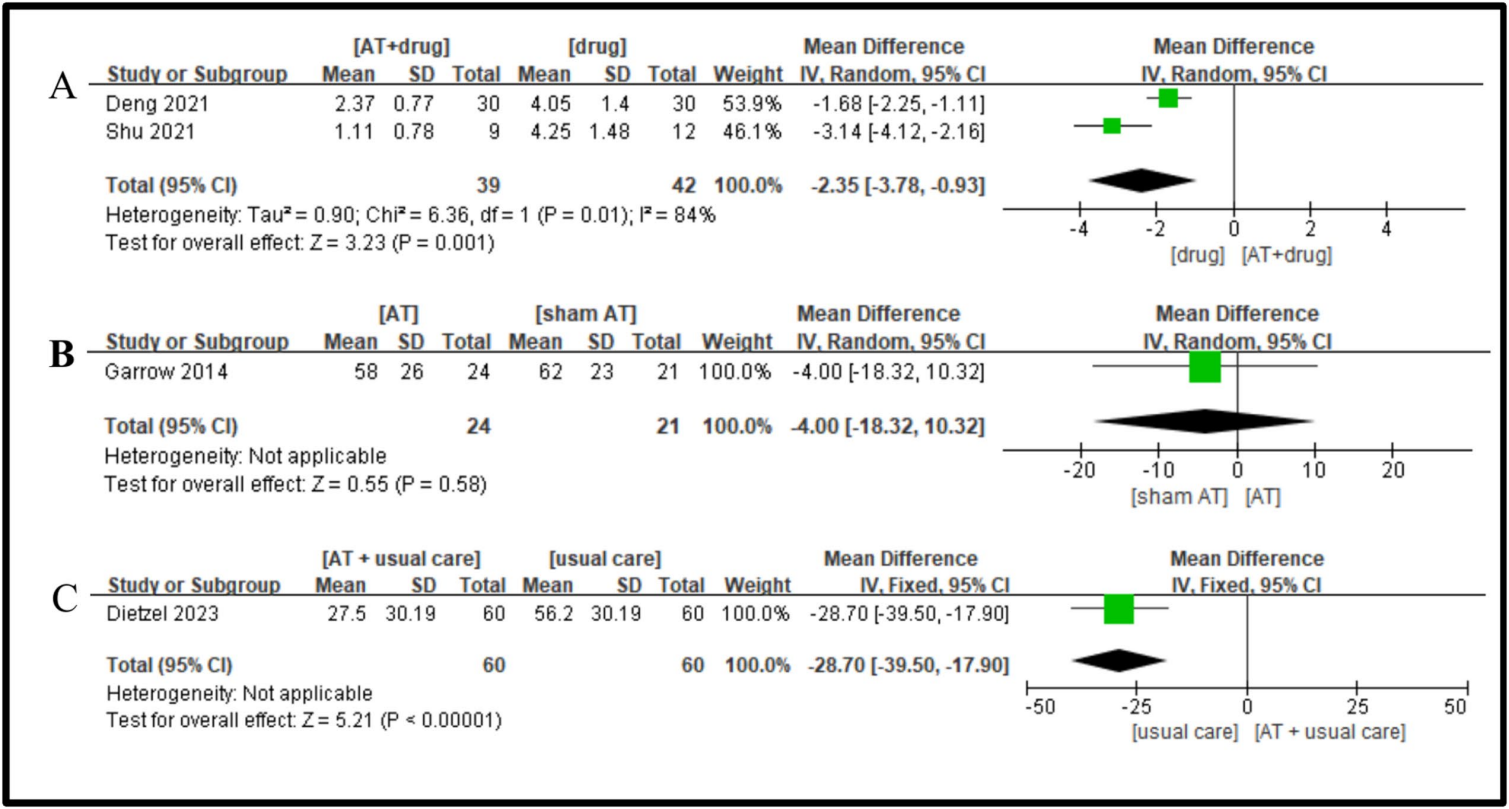
A Forest plot for VAS according to the comparison of **(A)** AT plus Drug vs. Drug, **(B)** AT vs. sham AT, **(C)** AT plus usual care vs. usual care.

Two studies were included (30, 35), and the heterogeneity test showed a large heterogeneity between studies (I^2^ = 84%, p = 0. 01), and a random-effects model was adopted. Meta-analysis results showed that the acupuncture plus drug group protocol was superior to the drug group in improving VAS in DPN patients, and the difference was statistically significant (MD = −2.35, 95%CI = −3.78 ~ −0.93, Z = 3.23, *p* = 0.001, Figure 10A).

One study showed (18) that no statistically significant difference acupuncture group protocol and the sham-needle group in improving VAS in DPN patients (MD = −4.00, 95%CI = −18.32 ~ 10.32, Z = 0.55, *p* = 0.58, Figure 10B).

Only one study (17) met the inclusion criteria, and it was noted that the acupuncture plus conventional group protocol exhibited a statistically significant improvement in VAS for DPN patients compared to the conventional group (MD = −28.70, 95%CI = −39.50 ~ 17.90, Z = 5.21, *p* < 0.00001, Figure 10C).

#### 3.4.9 Symptom scores

**Figure 11:**
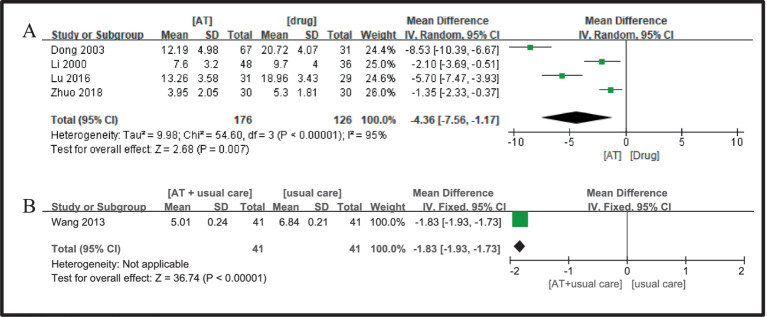
A Forest plot for symptom scores according to the comparison of **(A)** AT vs. Drug, **(B)** AT plus usual care vs. usual care.

Four studies (23, 27, 31, 34) were selected, showing significant heterogeneity (I^2^ = 95%, *p* < 0.00001), which led to the use of a random-effects model. The Meta-analysis results showed that the Meta-analysis results showed that the acupuncture group was better than the drug group in improving the symptom score of DPN patients (MD = −4.36, 95%CI = −7.56 ~ −1.17, Z = 2.68, *p* = 0.007, Figure 11A).

Only one study (28) was deemed eligible for inclusion, and the comparative analysis outcomes revealed that the acupuncture plus conventional group protocol was more effective than the conventional group in enhancing VAS in DPN patients, with a statistically significant difference observed (MD = −1.83, 95%CI = −1.93 ~ −1.73, Z = 36.74, *p* < 0.00001, Figure 11B).

#### 3.4.10 Total adverse effects

A total of 47 adverse reactions were reported in the four included studies (20, 30, 33, 36), including 2 cases of needle sickness, 18 cases of small hematomas, 1 case of localized swelling, 6 cases of pain, 1 case of itching, 7 cases of transient paresthesia, 1 case of cramps, 4 cases of transitory intensifying of DPN-related symptoms, 2 case of Mild dizziness, 1 case of chest pain, and 6 cases of tiredness.

#### 3.4.11 Sensitivity analysis

**Figure 12:**
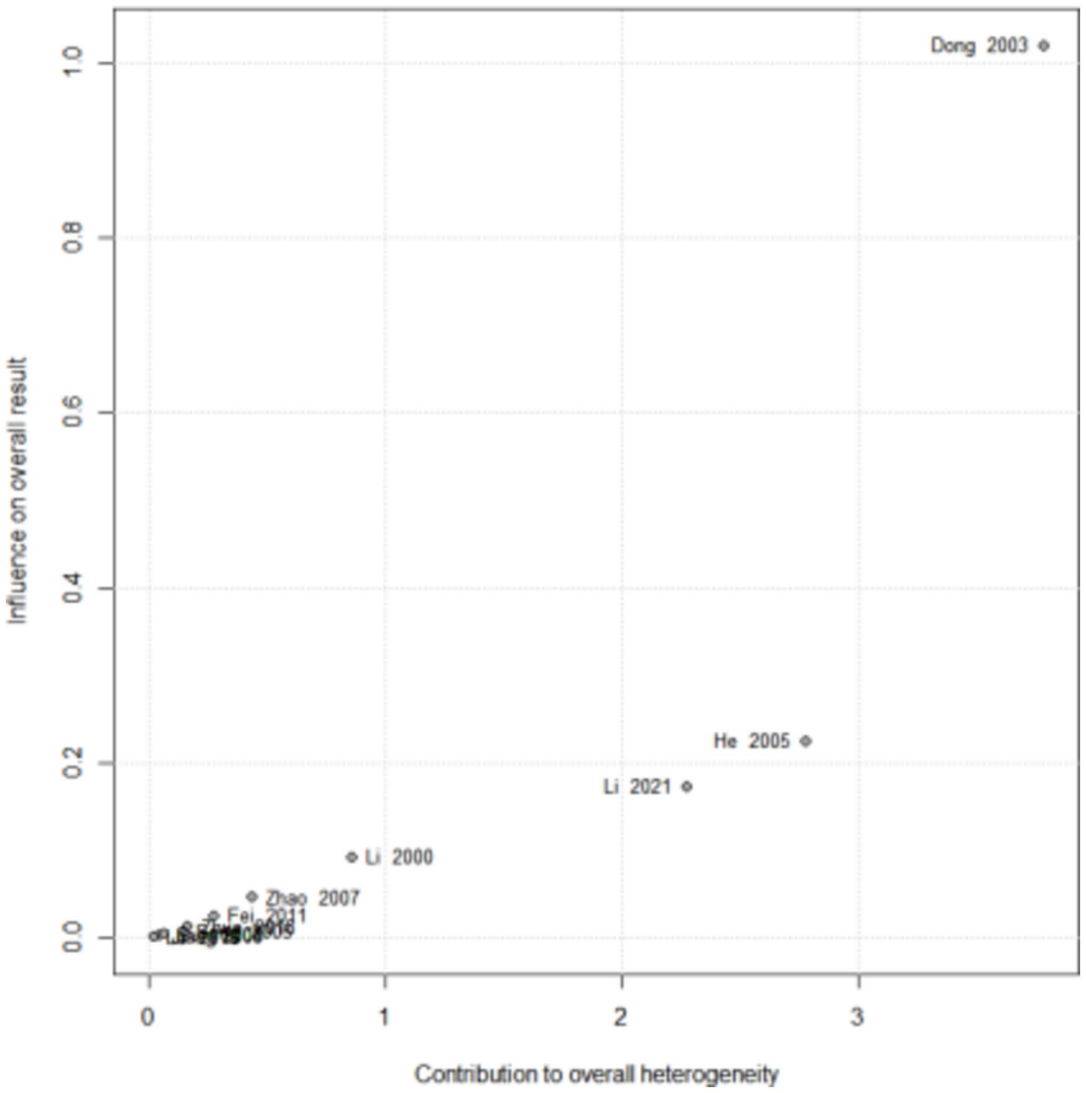
Baujat plot for total effective rate according to the comparison of (A) AT vs. Drug.

The total clinical effective rate is an important indicator of clinical efficacy. Therefore, a sensitivity analysis was conducted on the effective rate results for the acupuncture and drug groups that included the largest amount of data. By excluding studies individually, there was no significant change in the pooled effect size of the effective rate. From the results of Baujat chart, we found that three studies (21, 27, 31) led to the heterogeneity of Baujat chart results (Figure 12).

**Figure 13:**
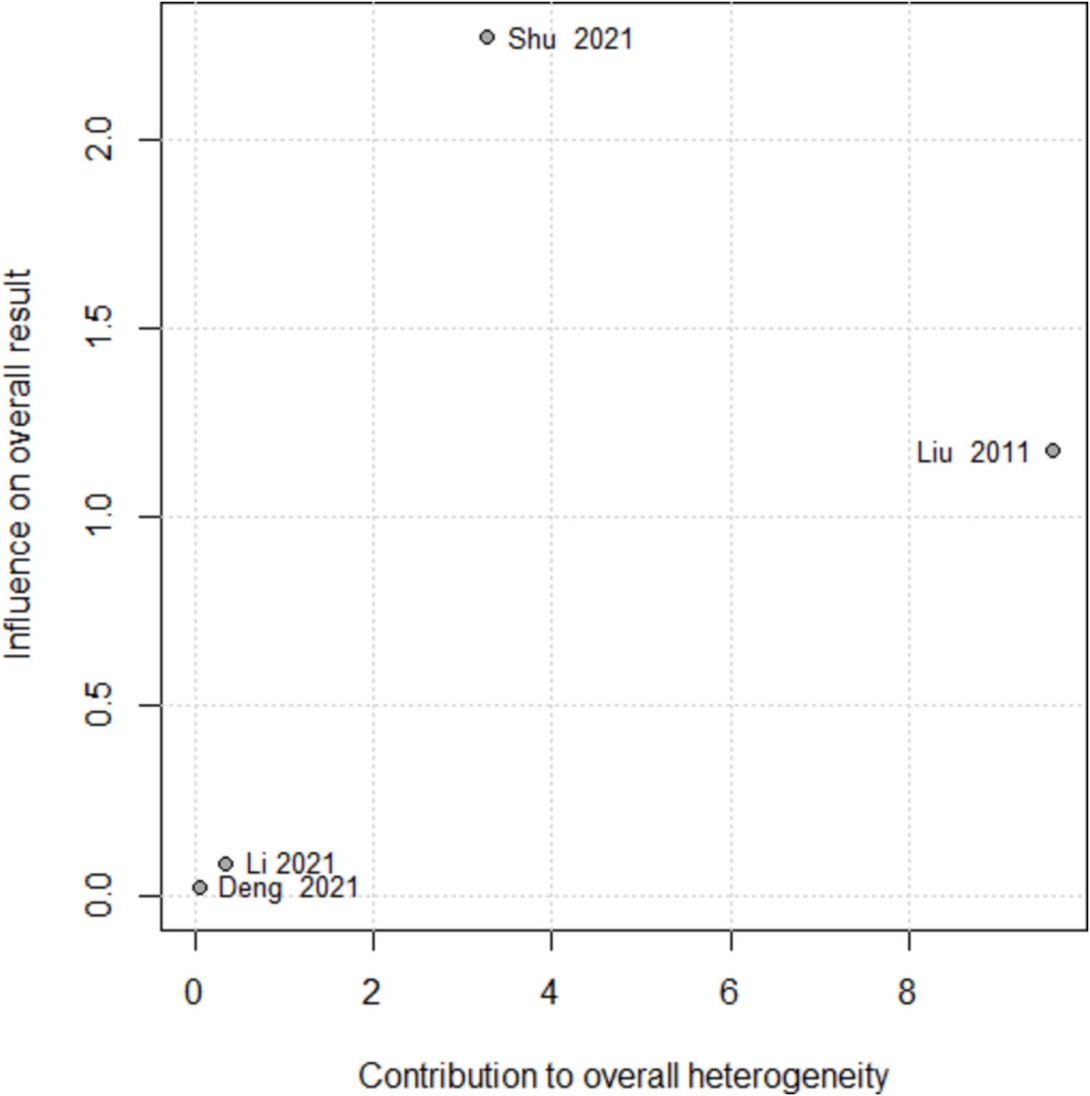
Baujat plot for total effective rate according to the comparison of AT plus Drug vs. Drug.

**Figure 14:**
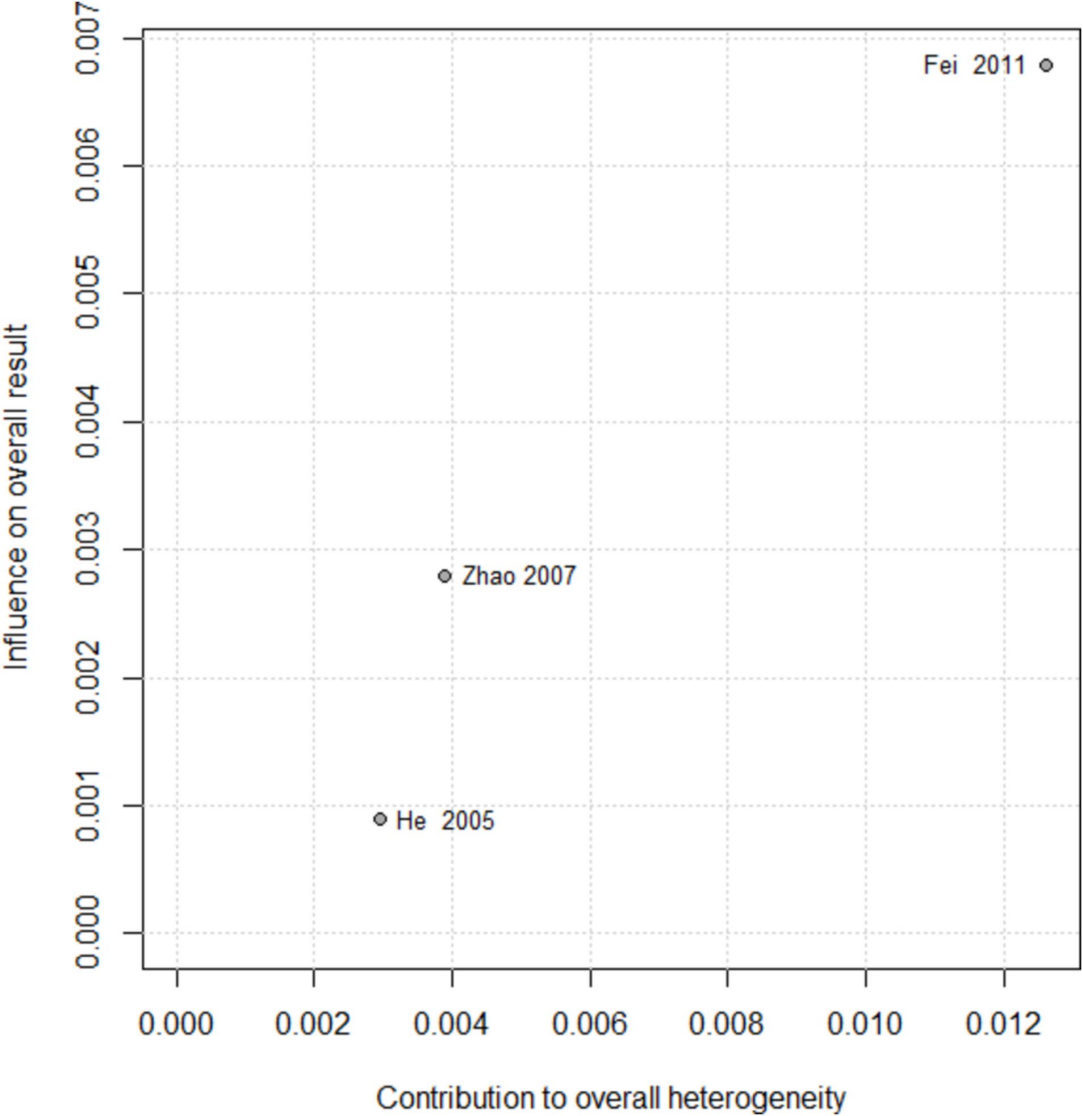
Baujat plot for MNCV in tibial nerve according to the comparison of AT plus Drug vs. Drug.

As regards the high heterogeneity found in the comparison of acupuncture with drug vs. drug on effective rate (I^2^ = 77%) and acupuncture vs. drug on MNCV in tibial nerve (I^2^ = 93%), we performed the sensitivity analysis. By excluding studies individually, there was no significant change in the pooled effect size of the effective rate, but an apparently weak decrease in heterogeneity was observed when one study was excluded (22). Moreover, from the results of the Baujat plot, we found that two studies (22, 30) unduly influenced heterogeneity as well as the pooled effect of the MNCV in tibial nerve (Figure 13). In the sensitivity analysis of nerve tibialis, the results showed that an apparently weak decrease in heterogeneity was observed when one study was excluded (32), and there were two studies (32, 33) that contributed overly to the heterogeneity from the results of the Baujat plot (Figure 14).

#### 3.4.12 Bias

**Figure 15:**
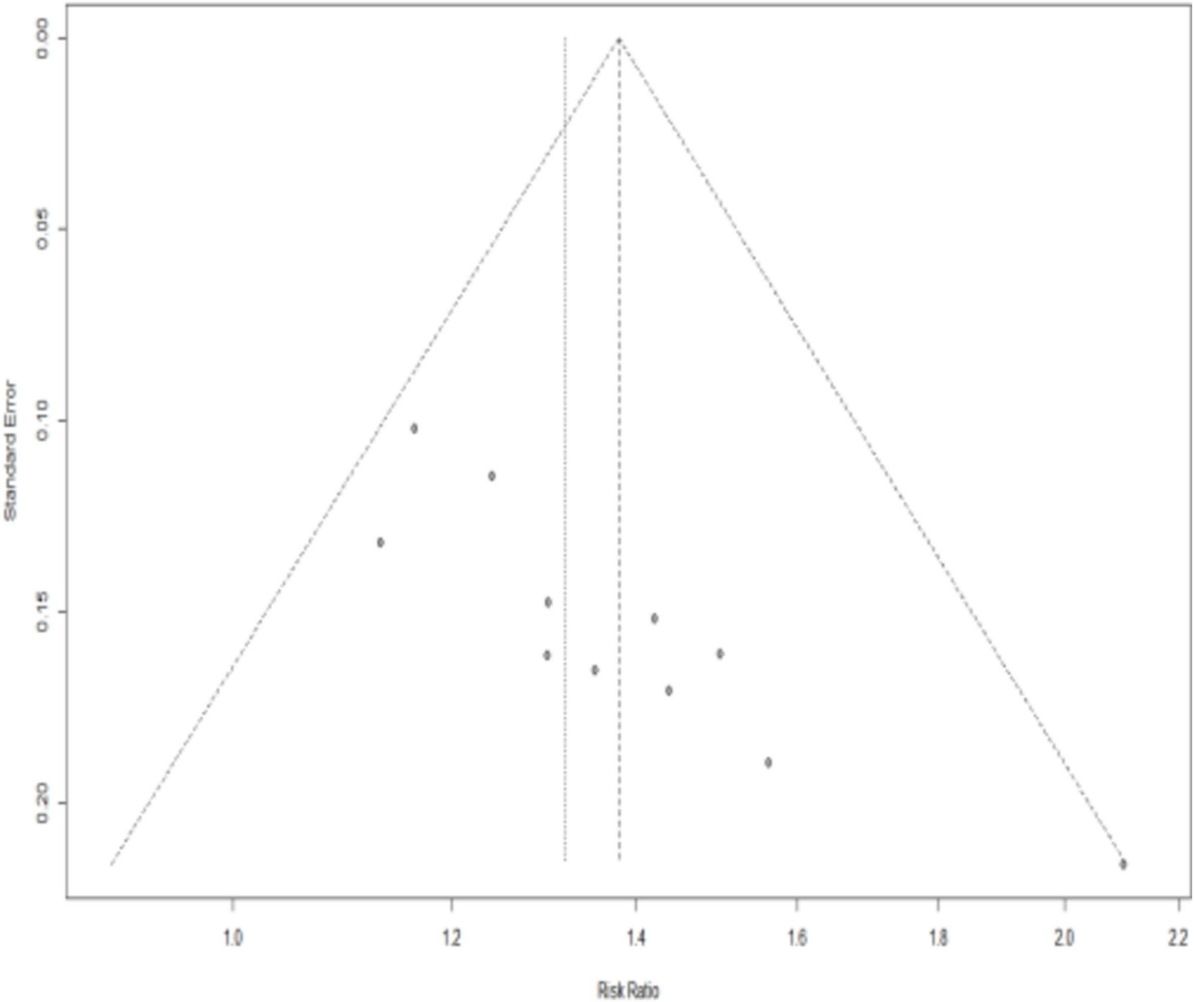
Funnel plots for the total effective rate.

We drew the funnel plot (Figure 15) and used Peters’ test (*t* = −1.40, *p* = 0.1955) to calculate the outcome of the total effective rate, which indicated no publication bias. However, publication bias in the outcome of effective rate may exist due to the asymmetrical funnel distribution and Egger’s test (*t* = 5.26, *p* = 0.0005). The trim-and-fill method showed that it was necessary to fill four potential unpublished studies in the funnel plot (Figure 1). A meta-analysis was re-performed for all the studies, results show a heterogeneity test is low (Q = 20.18, *p* = 0.1065), using fixed effect model, The combined results of the effect indicators (RR = 1.2464, 95%CI = 1.1498 ~ 1.3511, *p* < 0.0001) did not change significantly, indicating that the results were still statistically significant, with no reversal, so the combined results were robust (Figure 1).

**Figure 16:**
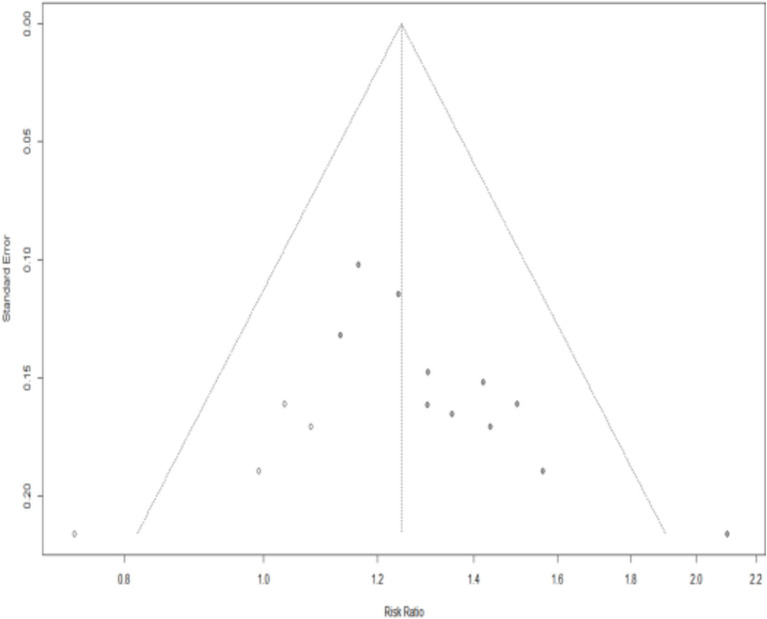
Funnel plot plotted after application of the trim-and-fill method.

## 4 Discussion

Our review aims to evaluate and refine the evidence from recent randomized controlled trials on acupuncture for the treatment of diabetic peripheral neuropathy. When comparing acupuncture with medication, conventional therapy, and sham acupuncture, our findings suggest that acupuncture is more effective in treating DPN and in improving nerve conduction velocity. Additionally, the combination of acupuncture and medication demonstrates a more significant improvement in nerve conduction velocity compared to medication alone. The combination of acupuncture and usual care improves DPN symptoms more effectively than usual care. In this meta-analysis, all included trials used acupuncture as a treatment option, and the clinical use of acupuncture for the treatment of DPN is varied, such as acupuncture, electroacupuncture, acupoint injection, and warm acupuncture. It can be seen that physical stimulation of acupoints is a safe and effective treatment for the potential treatment of diabetic peripheral neuropathy, and acupoints are an important basis for the efficacy of treatment, such as BL18, BL20, BL23, BL25, BL60, GB30, GB34, SP6 and ST36 are the most commonly used acupoints in the literature included in this study.

Acupressure is an external Chinese medicine treatment, and both acupressure and acupuncture use physical stimulation of acupoints to achieve therapeutic effects. Therefore, these two therapies have similar mechanisms of action and clinical efficacy. According to a previous review published by Fu et al. (37), the combination of herbal foot bath and acupressure therapy effectively improved sensory nerve conduction velocity (SNCV), motor nerve conduction velocity (MNCV), overall efficacy rate and neuropathy syndrome score compared with various types of control groups, such as Western medicine, oral Chinese medicine, other Western symptomatic treatments and blank control, and there were no case reports of adverse effects. The results of this trial are consistent with the findings of this study, which suggest that herbal footbath combined with acupressure may be safer and more effective in the treatment of DPN. In contrast to previous systematic reviews, we identified 20 new randomized controlled trials (17–36) and successfully assessed the treatment evidence.

Acupuncture, as a special therapy of Chinese medicine, has a wide range of indications, significant efficacy, safety and no side effects, and this review will identify the advantages and possibilities of using acupuncture in the treatment of DPN. In the early stages of DPN, the main manifestation is abnormal sensation in the limbs, which is distributed like a sock or glove, accompanied by numbness, pins and needles, burning, ants and ants, coldness, or as if stepping on a cotton pillow (38). This is followed by pain in the limbs, which is vague, tingling or burning, and is worse at night and during the cold period. In advanced stages, clinical manifestations of motor nerve disorders occur, such as hypotonia, muscle weakness to myasthenia and paralysis. Therefore, screening scales such as electromyography (EMG) for sensory nerve conduction velocity (SCV) and motor nerve conduction velocity (MCV) (39), Michigan Diabetic Neuropathy Score (MDNS) (39) and Toronto Clinical Scoring System (TCSS) (40) are important indicators of the patient’s condition. In a comparison of acupuncture with pharmacological therapies, the evidence showed that acupuncture had a significant effect on increasing treatment efficiency and was effective in improving sensory and motor nerve conduction velocities in the tibial and median nerves. Acupuncture was more effective than oral medication in relieving pain symptoms and improving quality of life in patients with painful diabetic peripheral neuropathy. Acupuncture showed better results than neurotrophic agents in improving circulation and significantly reducing clinical symptoms in patients with diabetic peripheral neuropathy of the lower extremities. The combination of acupuncture and medication was more beneficial than medication alone in improving nerve conduction velocity.

The pathogenesis of DPN is highly complex. Current research suggests that it is primarily caused by metabolic disorders resulting from hyperglycemia, dyslipidemia, and insulin resistance. These disorders include abnormal glycolytic pathways (41), increased advanced glycation end products (AGEs) (42), and alterations in protein kinase C signaling pathways. These metabolic disturbances further enhance oxidative stress (43) and inflammatory responses (44), leading to endoplasmic reticulum stress, mitochondrial dysfunction, DNA damage, and inflammation, collectively contributing to the onset of DPN. There is a certain connection between the pathogenesis of DPN and the therapeutic mechanism of acupuncture. Acupuncture may exert its therapeutic effects by regulating the pathophysiological processes of DPN. Studies have shown that the mechanisms by which acupuncture treats DPN may include the regulation of neurotrophic factor expression, such as nerve growth factor (NGF) and calcitonin gene-related peptide (45). Additionally, acupuncture improves glycolipid metabolism, such as reducing the accumulation of advanced glycation end products (AGEs) (46), and inhibiting the secretion of inflammatory mediators, such as interleukin-6 (IL-6), interleukin-8 (IL-8), and tumor necrosis factor-*α* (TNF-α) (47).

However, the analyses of acupuncture for DPN did not show a significant combined effect, partly due to the limited number of trials included. Therefore, the role of acupuncture in the treatment of DPN needs to be further investigated future. We also observed that more acupuncture sessions may introduce some heterogeneity, possibly due to increased reporting bias and poor compliance with long-term protocols. Screening scales such as TCSS and MDNS scores were significantly lower after acupuncture treatment than before treatment, and compared with drug therapy treatment before and after treatment changes plus significant, Chinese medicine symptom scores and symptom scores were significantly lower goodness to increase the effectiveness of treatment, acupuncture in combination with drugs and conventional therapies is more favorable than drugs alone. Furthermore, the incidence of adverse effects was significantly lower with acupuncture compared to drug therapy.

Based on our assessment, there is a high risk of bias in most of the included studies, which could lead to false positives, especially for subjects and staff blinded to an assessment. Blinding is difficult due to the specific nature of acupuncture therapy, where needles need to penetrate the skin and take time to remain there, whereas medications are taken orally as prescribed, where patients can easily distinguish whether they are receiving needles or medications, and where practitioners need to physically manipulate the patient’s skin to know the location and depth of the needles when performing acupuncture. Currently, the placebo needle methods commonly used in the design of acupuncture clinical trials include: acupoint/non-acupoint dermal surface needling, non-acupoint deep puncture needling, non-acupoint shallow puncture needling, specific acupoint dermal surface set of overlapping blunt-tipped needles, simulated dermal surface electrical stimulation, laser needling, and so on, but they all have certain limitations (48), and there is no accepted more mature method of placebo needling that can simulate the sensation of needle puncture without producing a therapeutic effect, which results in The experimental design of clinical trials of acupuncture is difficult to achieve strict blinded control. In addition, the clinical trials of acupuncture that have been conducted to date have not been able to blind the practitioners (49). Of the randomized controlled trials included in this review, only three trials (18, 20, 36) compared acupuncture with sham acupuncture. Two trials found that acupuncture was more effective than sham acupuncture in improving nerve conduction velocity. Another trial found that acupuncture was well tolerated and had no significant side effects. However, because of the differences in the outcomes between the two trials, they could not be analyzed together. So the placebo effect of acupuncture needs to be investigated further. There are also some limitations to this review. Firstly, many of the included trials had an unclear risk of bias, and most of the literature did not describe the specific method of random allocation and whether or not allocation was concealed. Second, despite the large number of randomized controlled trials, the outcome measures were very heterogeneous, which prevented large-scale meta-analyses. As a result, the level of evidence was mostly low or very low. Finally, most of the literature does not mention follow-up, and long-term effectiveness is more difficult to assess.

In the future, studies of acupuncture for diabetic peripheral neuropathy should be more rigorously designed and focusing on randomized methods, allocation concealment, blinding, selection of objective and comprehensive outcome indicators, and long-term follow-up to provide high-level clinical evidence. Clinical trials with a large sample size should also be carried out to clearly demonstrate the benefits of acupuncture and to provide robust evidence for clinical decision making. In addition, further studies should be conducted to provide guidelines for the clinical use of acupuncture for diabetic peripheral neuropathy in terms of acupuncture points, duration, frequency and treatment cycles.

In conclusion, acupuncture has the potential to be used as a routine treatment for diabetic peripheral neuropathy. Acupuncture has been shown to have better outcomes and fewer adverse effects than conventional Western medicine. The combination of acupuncture and pharmacological therapy is superior to pharmacological therapy alone for diabetic peripheral neuropathy. However, the level of evidence is low due to a high risk of bias and small sample sizes. To obtain high-quality and comprehensive evidence, future data from more rigorous clinical trials are needed. In addition, acupuncture has demonstrated better clinical outcomes for other complications of diabetes, such as diabetic nephropathy, diabetic foot, diabetic bladder and diabetic retinopathy, and has played an integrative role in improving the subsequent quality of life of diabetic patients.

## Data Availability

The original contributions presented in the study are included in the article/Supplementary material, further inquiries can be directed to the corresponding author.
