# Supplementary material for: The efficacy of acupuncture for diabetic peripheral neuropathy: a systematic review and meta-analysis of randomized controlled trails
Source: Front Neurol. 2024 Dec 20;15:1500709. doi: 10.3389/fneur.2024.1500709 (PMC11697586; doi:10.3389/fneur.2024.1500709)
Supplement: Supplementary file 1 [file Table_1.DOCX]

Supplementary Material

The efficacy of acupuncture for diabetic peripheral neuropathy: A systematic review and meta-analysis of randomized controlled trails.

# 1 Supplementary **Table**

**Table 1.** The detailed search strategy.

**Search strategy for CNKI**

TKA=('针灸'+'针刺'+'火针'+'艾灸'+'电针'+'梅花针'+'耳穴'+'穴位'+’针’+’灸’+’穴’) AND TKA=('糖尿病周围神经病'+'糖尿病周围神经炎'+'糖尿病周围神经痛'+'糖尿病足') AND TKA=('随机'+'对照'+'试验')

**Search strategy for Wanfang**

( 摘要:(针灸) or 摘要:(针刺) or 摘要:(电针) or 摘要:(艾灸) or 摘要:(穴位) or 摘要:(耳穴) or 摘要:(火针) or 摘要:(梅花针) or 摘要:(针) or 摘要:(灸) or 摘要:(穴)） and （ 摘要:(糖尿病周围神经病变) or 摘要:(DNP) or 摘要:(糖尿病周围神经炎) or 摘要:(糖尿病周围神经痛) or 摘要:(糖尿病足)）

**Search strategy for VIP**

R=(针灸 OR 针刺 OR 火针 OR 艾灸 OR 电针 OR 梅花针 OR 耳穴 OR 穴位 OR 针 OR 灸 OR 穴) AND R=(糖尿病周围神经病 OR 糖尿病周围神经炎 OR 糖尿病周围神经痛 OR 糖尿病足) AND R=(随机 OR 对照 OR 试验)

**Search strategy for PubMed**

((acupuncture) OR (lectroacupuncture) OR (Fire needle) OR (plum blossom needle) OR (moxibustion)) OR (acupoint) OR (auricular acupuncture)) AND ((peripheral nervous system diseases) OR (diabetic peripheral neuropathy) OR (DPN) OR (DSPN)) AND ((randomized controlled trial) OR (RTC) OR (random) OR (blind) OR (control))

**Search strategy for Web of Science**

(ALL=(acupuncture) OR ALL=(lectroacupuncture) OR ALL=(Fire needle) OR ALL=(plum blossom needle) OR ALL=(moxibustion) OR ALL=(acupoint) OR ALL=(auricular acupuncture)) AND (ALL=(peripheral nervous system diseases) OR ALL=(diabetic peripheral neuropathy) OR ALL=(DPN) OR ALL=(DSPN)) AND (ALL=(randomized controlled trial) OR ALL=(RTC) OR ALL=(random) OR ALL=(blind) OR ALL=(control))

**Search strategy for Cochrane Library**

| Search | Terms |
| --- | --- |
| #1 | (ao.puncurejt,ab,kw OR fecroscupuncturek,ab,kw OR (Fire needeyil,ab,.kw OR iphm blssom needlejilab,kw OR (morbustonrt ab kw |
| #2 | (ac.pointa,ab how oit (a.ricular ac.puncnure)sil. ab,low |
| #3 | #1 OR #2 |
| #4 | lperipheral nervous syslem diseases ktl,ab,kw OR (dlabetl peripheral neuropathya,ab ,kw OR (DPN)a,ab kw |
| #5 | dspn |
| #6 | #4 OR #5 |
| #7 | (randomized coniralled trial;tl,ab,hkw OR (RcTyt,ab,kw Of (randomyti,ab, kw OR (bindyti, ab, kw OR (contrelyti, ab.lkw |
| #8 | (dyssomnia):ti,ab,kw OR (sleep disorders):ti,ab,kw OR (sleep disturbance) :ti,ab,kw OR (sleep dysfunction):ti,ab,kw OR (sleep):ti,ab,kw OR (sleepiness):ti,ab,kw OR (sleep problem):ti,ab,kw OR (insomnia):ti,ab,kw OR (restless legs syndrome):ti,ab,kw OR (rapid eye movement sleep behavior disorder):ti,ab,kw OR (excessive daytime sleepiness):ti,ab,kw OR (obstructive sleep apnea):ti,ab,kw OR (circadian rhythm):ti,ab,kw |
| #9 | #3 AND #6 AND #8 |

**Search strategy for Cinahl**

| Search | Terms |
| --- | --- |
| #1 | acupuncture OR lectroacupuncture OR Fire needle OR plum blossom needle OR moxibustion OR acupoint OR auricular acupuncture |
| #2 | peripheral nervous system diseases OR diabetic peripheral neuropathy OR DPN OR DSPN |
| #3 | randomized controlled trial OR RCT OR random OR blind OR control |
| #5 | #1 AND #2 AND #3 |

**Search strategy for AMED**

| Search | Terms |
| --- | --- |
| #1 | acupuncture OR lectroacupuncture OR Fire needle OR plum blossom needle OR moxibustion OR acupoint OR auricular acupuncture |
| #2 | peripheral nervous system diseases OR diabetic peripheral neuropathy OR DPN OR DSPN |
| #3 | randomized controlled trial OR RCT OR random OR blind OR control |
| #5 | #1 AND #2 AND #3 |

# 2 Supplementary Figure 1

**Figure 1. Sensitivity analysis for studies reporting on the effective rate results for the acupuncture and drug groups .**


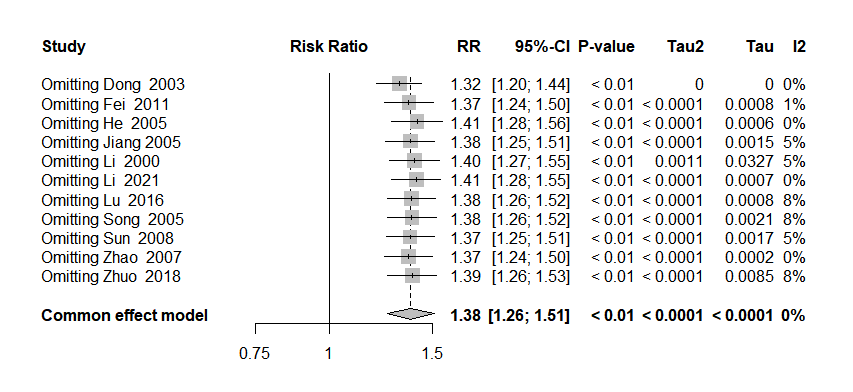


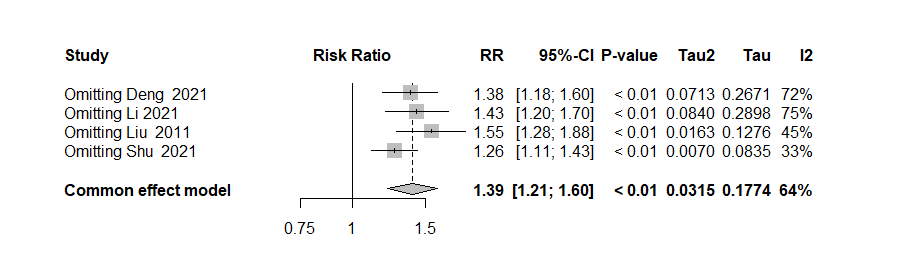
**Figure 2. Sensitivity analysis for studies reporting on on the effective rate results for the acupuncture plus drug and drug groups .**


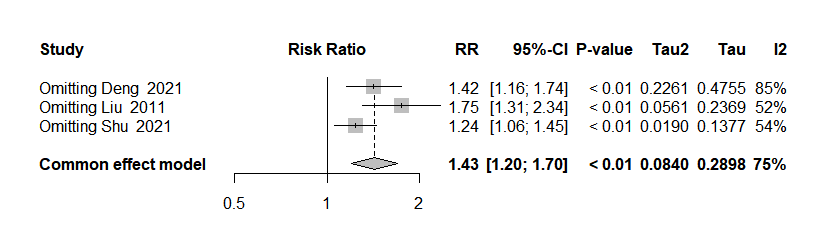
**Figure 3. Sensitivity analysis for studies reporting on MNCV in tibial nerve for the acupuncture and drug groups .**
